# Results of an Action-Research on Epilepsy in Rural Mali

**DOI:** 10.1371/journal.pone.0044469

**Published:** 2012-08-31

**Authors:** Elisa Bruno, Karamoko Nimaga, Ibrahima Foba, Philippe Vignoles, Pierre Genton, Ogobara Doumbo, Daniel Gérard, Pierre-Marie Preux, Guy Farnarier

**Affiliations:** 1 Institut National de la Santé et de la Recherche Médicale (INSERM) U1094, Tropical Neuroepidemiology, University of Limoges, School of Medicine, Institute of Tropical Neurology, Centre Hospitalier Universitaire (CHU), Limoges, France; 2 Department Gian Filippo Ingrassia, Section of Neurosciences, University of Catania, Catania, Italy; 3 Association des Médecins de Campagne/Réseau Action-Recherche sur l’Epilepsie (RARE) Network, Bamako, Mali; 4 Department of Epidemiology of Parasitic Diseases, Faculty of Medicine, Bamako, Mali; 5 Santé-Sud Non-Governmental Organization, Bamako, Mali and Marseille, France; 6 Access to Drugs Department, Sanofi-Aventis, Paris, France; 7 Department of Clinical Neurophysiology, Community Health Research Unit (CHRU) Hôpital Nord and Faculty of Medicine/Aix-Marseille University, Marseille, France; California Pacific Medicial Center Research Institute, United States of America

## Abstract

**Purpose:**

To evaluate the RARE (Réseau Action-Recherche sur l’Epilepsie) program, a model of managing and treating people with epilepsy (PWE) at a primary health-care level in rural areas of Mali, we assessed treatment efficacy and compliance of patients who underwent the first year follow-up.

**Methods:**

A network of rural general practitioners (GPs) settled in six rural districts of the regions of Koulikoro, Segou and Sikasso, was involved in the diagnosis, evaluation and monitoring of all the identified PWE and in the distribution of phenobarbital (PB). All the participants were included in a prospective database and followed-up by GPs at 4 months intervals during the first year. Seizure frequency, treatment doses and appearance of adverse events (AEs) were systematically recorded. Efficacy was evaluated in terms of reduction of seizures frequency while noncompliance in terms of time to study withdrawal for any cause.

**Key findings:**

596 patients treated with PB were included in the analysis. Of these, 74.0% completed the first year follow-up. At the final visit, 59.6% were seizure-free: 31.0% for 12 months, 10.2% for 8 months and 18.4% for 4 months. Adults and patients with convulsive seizures were the most drug-resistant (p<0.002). Few AEs were recorded. The multivariate analysis showed that being a woman, presenting convulsive seizures, having more than 5 seizures/month and had never be treated were predictors of withdrawal (p≤0.05) at 12 months.

**Significance:**

This study showed a good response and compliance to the treatment and allowed the identification of some factors associated with failure of management in a setting very near to clinical practice. Awareness campaigns are needed to assure a broader accessibility to treatment and to improve the compliance and continuity with treatment programs.

## Introduction

Epilepsy is one of the most common worldwide neurological disorder that could be effectively treated, representing a significant proportion of treatable burden of disease [1]. The World Health Organization (WHO) suggested that medical disorders requiring a low technological approach should be managed at a primary health-care level [2]. This of course could be applied to the management of epilepsy. Moreover, especially in rural areas, a continuous local medical monitoring aimed at promoting health education and at assuring a good compliance is required to obtain a good response to treatment and thus the success of health programs. Unfortunately, in resource-poor countries the limited medical means, infrastructure and the lack of health personnel represent some of the main existing obstacles to the promotion of such local health-care strategies [3], [4]. Actually, it was estimated that the neurologist-to-population ratio in sub-Saharan Africa is 3/10,000,000 [5] while, the ideal ratio should be 1 per 100,000 [6], [7]. Furthermore, nearly two-thirds of people in resource-poor countries reside in rural areas, whereas all the neurologists practice in or close to big cities and towns [8]. Therefore people with epilepsy (PWE) encounter several obstacles to adequate treatment that lead a significant proportion of them to be untreated or to receive only traditional remedies [1], [6], [9]. The promotion of local health-care system and the training of experts in epilepsy were the main objectives pursued from 1996 to deal with epilepsy in Mali. The cooperation between the AMC (Association des Médecins de Campagne), a group of general practitioners (GPs) settled in rural areas, and Santé-Sud, a nongovernmental organization (NGO), gives rise to a comprehensive, self-sustained program called the RARE: Réseau Action-Recherche sur l’Epilepsie, Research-action network on epilepsy [10]. The aim of this network was to provide practical and efficient tools for the management of PWE located in the area and to promote research activities. The first phase of the project provided an anthropological study on epilepsy which took place in both urban and rural setting [11], [12], [13]. A door-to-door epidemiological survey was performed in May 1998 to identify PWE. The screening of 5,243 individuals living in 18 villages around the area of Bamako permitted the detection of a prevalence of epilepsy of 13.35 per 1,000 [14], [15]. Starting from 1999, six volunteers of the AMC underwent an intensive training on epileptology and developed a protocol of treatment and management of epilepsy in the areas around the villages of Baguineda and Tyenfala. The RARE Franco-Malian team was responsible for the organization of workshops and for the control of practical aspects, including the delivery of antiepileptic drugs (AEDs) [16], [17], [18]. From January 1999 to January 2000, a one-year pilot study, based on regular domiciliary monitoring of each patient by trained rural GPs and on adequate AEDs supplies, showed a good efficacy and an excellent level of compliance achieved on 96 patients successfully treated with free phenobarbital (PB): 80% of patients were seizures-free for at least 5 months, and a further 16% had experienced a substantial reduction in seizures frequency. There were very few adverse events (AEs) and no case of overdosing was reported [18]. Therefore, in 2003 the domiciliary monitoring was stopped and the treatment program was expanded to all PWE requiring assistance to their health-care centers, creating a treatment setting very close to clinical practice. To evaluate this case management model developed at a community based level, we assessed program efficacy and compliance at first year follow-up for patients who started treatment between December 2003 and December 2005.

## Methods

### Study Setting

The project was carried out in 34 villages, most of them along the river Niger, in the regions of Koulikoro, Segou and Sikasso, where the RARE developed a model of epilepsy care aimed at identifying, diagnosis, treating and following-up people with active epilepsy in rural areas. The number of inhabitants of the rural area was about 90,573 according to the administrative electoral census of 2001 (RACE Recensement Administratif à Caractère Electoral). The villages are situated in the sahelian savanna, characterized by a north-sudanian tropical climate. The local economy is predominantly based on agriculture and animal breeding.

### Patients and Procedures

The GPs training was provided by a team of Franco-Malian physicians and by supervising neurologists. The module covered epidemiological aspects of epilepsy, causes, diagnosis and differential diagnosis, types of treatment and management aspects. After a week of initial training, the program provided additional workshops planned every six months and performed at local health facilities. Participants were asked to complete a questionnaire to ascertain knowledge and practice in relation to epilepsy both at the beginning and at the end of the first training session, in order to evaluate the acquired knowledge and develop the succeeding workshops. The coordination of the training was assigned to an ad-hoc trained physician of the district of Markacoungo, chosen as the “head” of the network. Six GPs were designate as responsible of six primary health-care centers located in six rural districts in the villages of Markacoungo, Baguineda, Diebe, Kourouma, N’Debougou and Konina. An educational campaign on epilepsy was launched in these areas, involving the trained GPs, paramedical and social workers, local authorities, traditional chiefs and teachers. The campaign was promulgated by radio broadcast, printed material and was supported by a team of health workers who held discussions in public places and schools in the local language. The aim was to sensitize the population on epilepsy and its treatment and to promote the presence of qualified personnel available at the local health-care centers. Thanks to this campaign, people living in these areas and suspected of having epilepsy started to come to consultation at the health-care centers and were subsequently screened for epilepsy and eventually enrolled in the treatment program. The GPs were responsible for the evaluation, diagnosis and management of all the identified PWE. Demographic data, medical history and clinical status were assessed and recorded for diagnostic confirmation. Diagnosis of epilepsy was based on clinical ground, examination and medical history. The criteria used for confirmation and classification of epileptic seizures were the 1981 International Classification of Epileptic Seizures [19]. All the identified PWE of all ages with active epilepsy, defined by the recurrence of two or more episodes of seizures in the last 2 years [20], underwent treatment. The study received approval from the ethical committee of the University of Bamako, Mali.

### Management and Follow-up

PWE entering the study were treated with generic PB monotherapy. Tablets were sold at production cost and left in the care of each patient. Starting PB doses were 50 mg for adults and 25 mg for children, taken once daily, and increased by amounts of 25/50 mg. All the participants were included in a prospective database and followed up at 4-months intervals over the first year of management. Patients attended their local clinic regularly for titration, doses adjustment and to receive further supplies of medication. The GPs completed a follow-up form at each visit, systematically recording seizure frequency, treatment doses and appearance of AEs.

Efficacy was evaluated in terms of reduction of seizures frequency while noncompliance in terms of time to study withdrawal for any cause. We considered all participants lost to follow-up as withdrawals.

### Statistical Analysis

Results for quantitative variables were expressed as means ± standard deviation (SD) while qualitative variables as frequencies and percentages. Categorial variables were compared using the chi-square test whereas the t test and the ANOVA were used to compare continuous variables. Time to first seizure was determined with a Kaplan-Meier survival analysis. Two groups were compared during the analysis: the group of “convulsive seizure”, consisting of PWE presenting convulsive seizures, including both generalized tonic-clonic (TC) seizures and secondary generalized TC seizures and the group of “non-convulsive seizures”, including other types of seizure (partial seizures without secondary generalization, absences). The log-rank test was used to assess statistical significance of between-group differences. Seizure-free rates at 4, 8 and 12 months were based on Kaplan-Meier survival analysis. The Cox proportional hazard model was used to assess the difference between groups and time to withdrawal for any cause, adjusting for demographic and clinical characteristics. Multivariate analysis was performed using logistic regression to analyse the withdrawals during the follow-up. Parameters associated with the outcome at the univariate analysis with a threshold of p = 0.10 were included in the model. Variables considered a priori confounders (age and sex) were entered in the multivariate module regardless the univariate analysis. The model was manually constructed using the likelihood ratio test (LRT) to compare the log likelihood of the model with and without a specific variable. The possible interaction was also evaluated by the LRT (test of violation of proportional odds). Significance level was fixed to p≤0.05.

## Results

### Characteristics of the Patients

**Figure 1:**
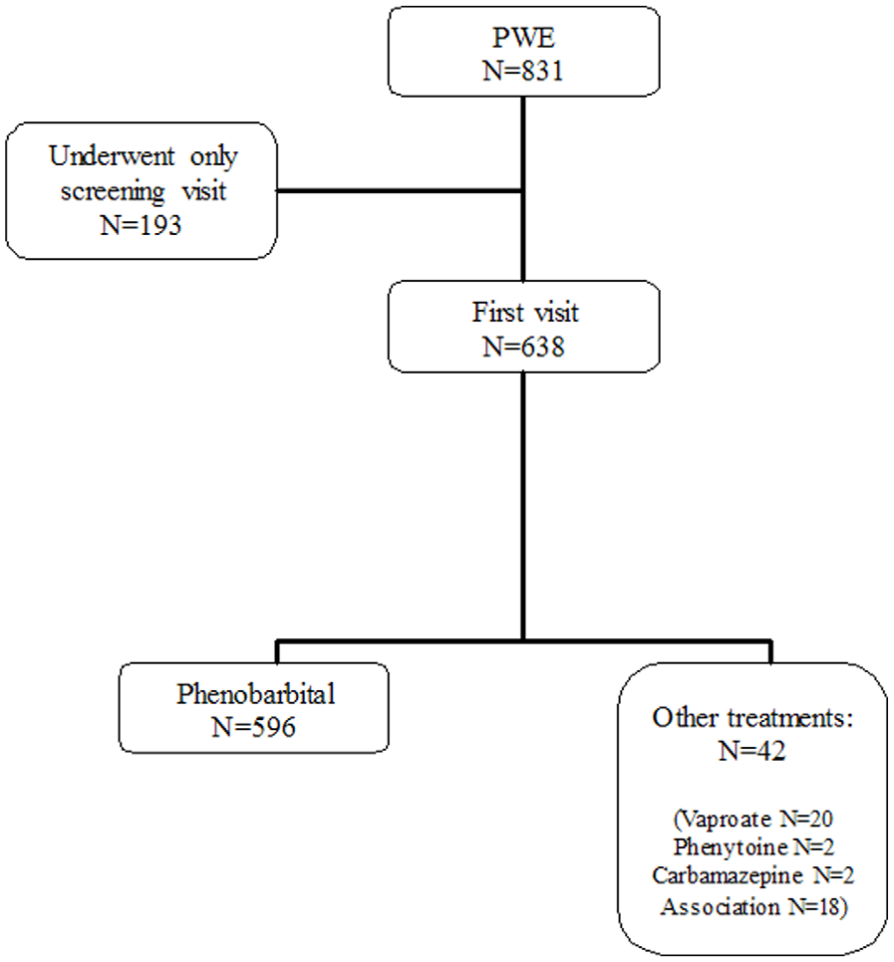
Patients enrollment and treatments underwent.

A total of 831 patients, who had consulted the rural GPs, satisfied the diagnostic criteria and were considered eligible for the study. One-hundred-ninety-three of them did not go beyond the screening visit. We focused the analysis on 596 patients who started treatment with PB, excluding an exiguous group of patients (42 people) treated with others medications (figure 1).

Baseline characteristics (age, sex, area) of the 596 patients were not significantly different from the characteristics of the 235 that were excluded from the analysis (p>0.1). Of the 596 patients, 383 (64.3%) were men and 213 (35.7%) were women (sex ratio 1.8; p = 0.0001). The mean age was 19.3 (±13.4) years for men and 19.8 (±11.9) years for women (p = 0.8), with a mean age at seizure onset of 11.2 (±11.7) years and a median of 3.5 seizures/month (IQR 3 seizures/month) before starting treatment. Demographic and clinical characteristics of the patients are summarized in Table 1.

**Table 1:** Characteristics of the 596 patients.

Characteristics	n	%
**Area**		
Baguineda	60	10.1
Diebe	68	11.4
Konina	44	7.4
Kourouma	56	9.4
Markacoungo	302	50.7
N'Debougou	66	11.1
**Occupation for adults (≥18 years; n = 322)**		
Farmer	119	37.0
Breeder	13	4.0
Fischerman	2	0.6
Merchant	18	5.6
Employee	1	0.3
Housewife	87	27.0
Unemployed	25	7.8
Other	57	17.7
**Marital status**		
Married	146	24.5
Unmarried	150	25.2
Children	274	46.0
Other	26	4.4
**Family history of epilepsy**		
Yes	178	29.9
No	390	65.4
Not know	28	4.7
**Past treatment with AEDs**		
Yes	154	25.8
No	325	54.5
Not know	117	19.6
**Past AEDs taken (n = 154)**		
Phenobarbital	92	59.7
Carbamazepine	7	4.5
Phenitoine	3	1.9
Others	11	7.1
Not know	41	26.6
**Past traditional treatments**		
Yes	538	90.3
No	21	3.5
Not know	37	6.2

### Classification of Epilepsy and PB Doses

According to the ILAE classification of 1981, 396 (66.4%) patients had generalised seizures, 49 (8.2%) partial seizures, 109 (18.3%) had partial seizures with secondary generalization and 42 (7.1%) were not classified.

PB daily maintenance dose, defined as that able to controlled seizures and being well tolerated, was 150–200 mg for adults and 50–100 mg for children.

### Withdrawals

**Figure 2:**
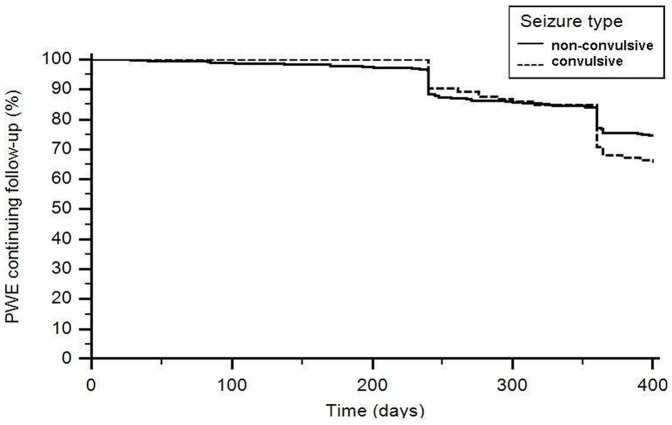
Kaplan-Meier plots illustrating time to withdrawal for any cause for the groups of convulsive and non-convulsive seizures.

The number of patients who underwent the 12-month follow-up was 441 (74.0%). Kaplan-Meier plots illustrating time to withdrawal for any cause during the follow-up are shown in figure 2. The difference between convulsive and non-convulsive seizures was not statistically significant when tested by the logrank test (p = 0.08), while Cox proportional hazard model adjusted by age, sex and seizure frequency showed a difference between the two groups (p = 0.01).

The logistic regression showed that be a woman (OR = 1.8; p = 0.03), presenting convulsive seizures (OR 1.6; p = 0.05), had never sought medical-care for epilepsy (OR = 2.1; p = 0.05), had never been treated with AEDs (OR = 1.8; p = 0.07) and presenting more than 5 seizures/month (OR 1.2; p = 0.03) were risk factors to be withdrawn at 12 months (table 2). Epilepsy remission at last visit seemed to not affect patients compliance (p = 0.7).

**Table 2:** Factors influencing patients withdrawal: univariate and multivariable analysis of the 596 treated people with epilepsy.

	Follow-up			
	Univariate		Multivariable	
	OR	p value	OR	p value
**Gender**				
Male	1.0		1.0	
Female	**1.4**	**0.1**	**1.8**	**0.03**
**Age group**				
Adults (>18 year)	1.0		1.0	
Children (<18 year)	**1.5**	**0.04**	1.1	0.7
**Epilepsy type**				
Non convulsive	1.0			
Convulsive	**1.6**	**0.04**	**1.6**	**0.05**
**Past medical consult**				
Yes	1.0			
No	**3.3**	**0.000**	**2.1**	**0.05**
**Past treatment**				
Yes	1.0			
No	**2.7**	**0.000**	**1.8**	**0.07**
**Seizure frequency**				
No seizure	1.0			
1/month	1.7	0.1	1.7	0.2
2/month	1.4	0.3	1.1	0.8
3–4/month	1.5	0.3	2.2	0.8
5/month	**1.1**	**0.06**	**1.2**	**0.03**
**Remission at last visit**				
Yes	1.0			
No	1.2	0.3	0.9	0.7

### Assessing Program Efficacy

The number of seizures/month registered at each follow-up visit showed an important reduction during the first year of treatment with PB. At their last visit, the percentage of PWE declaring no seizures passed from 0% to 59.6%, while the number of patients with 5 or more seizures/month passed from 26.3% to 3.6%, showing a reduction in seizure frequency. At their last observation, a total of 185 (31.0%) subjects were seizure-free for 12 months, 61 (10.2%) for 8 months while 110 (18.4%) for 4 months.

**Figure 3:**
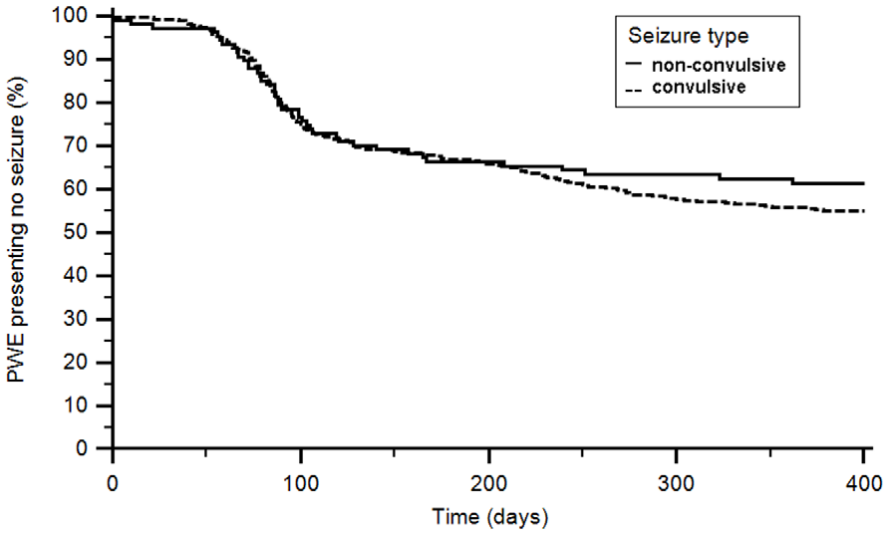
Time to first seizure. Kaplan-Meier analysis for cumulative event rates during the first year follow-up for the groups of convulsive and non-convulsive seizures.

Kaplan–Meier plots illustrating time to first seizure during the first year of treatment is shown in figure 3. Subjects who prematurely discontinued from the study were censored at their last visit. The difference between convulsive and non-convulsive seizures assessed with log-rank test was not statistically significant (p = 0.3). Cox hazard ratio model adjusted for age, sex and seizures frequency showed a difference between the groups (p = 0.001). The multivariable model of logistic regression adjusted for sex and seizure type, showed that adults (OR 1.7; p = 0.002) and patients presenting more than 5 seizure/month (OR 1.5; p = 0.000) were risk factors for presenting other attacks.

### Safety

A total of 104 patients (17.4%) reported AEs during the first month of treatment with PB, the main represented by sleepiness (12.4%) and hyperactivity (2.5%), followed by depression (1.7%), loss of balance (0.3%) and rash (0.2%). All AEs decreased over time.

## Discussion

The RARE program was aimed at implementing treatment and management of epilepsy in rural areas of Mali at a primary health-care level within the existing services. Given the dearth of physicians in countries such as Africa (<1/25,000 population) [21] and the distressing lack of neurologists [5], the process of “task shifting” to less specialized health workers has been encouraged by WHO [21] and has been applied with success in different countries worldwide [22], [23], [24], [25], [26]. The RARE first objective was the demonstration that an appropriate management of PWE could be achieved through the development of local staff performance whose background, language and social attributes made accessible to local population [27]. Improving medical presence in rural areas, based on first-line GPs is the best way to provide quality health-care especially for chronic diseases, such as epilepsy [27], [28], [29], [30].

In order to early highlight the possible weaknesses of the treatment program and to identify the factors associated with the failure of management, we focused our analysis on the data obtained during the first year of follow-up. After the identification of the main determinants of inefficacy and noncompliance, the subsequent local educational campaigns were designed with the attempt to overcome the emerging issues. From 2007, the RARE program designed and implemented a network aimed at finding solutions to practical problems encountered by both doctors and patients in rural areas. Special attention was given to the promotion of treatment compliance and to the involvement of women and children in the treatmetn program. Moreover, availability of others AEDs supplies was ensured to deal with drug-resistent patients.

Even if several data on PB treatment in developing countries are reported in literature, this is probably one of the few studies carried out in a very large cohort of PWE followed-up in a setting very close to clinical practice.

The first-line GPs based nature of the study has probably led to obtain a cohort of PWE with a significant majority of men. This finding should be attributed to the probably easier access to health facilities for men than for women. Male workers present easier possibility to displace and more economics resources that facilitate health-care seeking. The male over-representation has been observed in many studies carried out in the African continent [31], [32] and the sex ratio reported in our study (1.8) was similar to that reported for African adults [33]. The age at seizure onset pointed out in our survey appeared to be very low: <10 years old for 52.4% of patients. Most epidemiologic surveys suggest that age at onset for epilepsy in resource-poor countries is lower than in more rich regions [34]. Actually, the lack of local medical infrastructure and suboptimal ante-perinatal care, especially in rural areas, results in a greater exposure to birth injury, neonatal hypoxia, cerebral ischemia and acquired infections that constitute important risk factors for epilepsy.

The identification and classification of epilepsy was totally entrust to the GPs and, due to the absence of paraclinical investigations (EEG, neuroimaging) [35], was based on clinical methods alone. This could have lead to an overestimation of generalized TC seizures that predominated (55.7%). A large proportion of presentations was farther classified as partial seizures with secondary generalization (18.3%). On the contrary, pure partial seizures were less frequent.

Patients presenting more than 5 seizures/month were well represented (26.3%) in the cohort, as happens in hospital-based surveys based on clinical classification of seizures [33] and as a consequence of the lack of treatment in these regions [22].

Surprisingly in this population a high proportion of patients (25.9%) reported to have received AEDs in the past. Even if this percentage stands in the upper limit of the 6–26% range reported in others resource-poor countries [22], [36], it should be considered that the majority underwent only an intermittent self-medication.

The PB treatment protocol showed a good efficacy and tolerability during the follow-up. PB was chosen because of its low cost, its effectiveness in controlling seizures, its once daily administration and because its indication as first-line treatment for partial and generalized TC seizures in resource-poor countries [37]. During the follow-up there was a significant reduction in seizures frequency, and the percentage of seizure-free patients after one year follow-up was 59.6%. Similar studies using PB treatment showed comparable results: 53% seizure-free in Kenya [22], 52.4% in Tanzania [31], 56% in Malawi. Different findings were reported in other countries outside Africa as in China where 33% of patients were seizure-free after 1 year [37] and India, where the percentage of seizure-free patients ranged from 58 to 66% [38]. Epilepsy type was analysed as a binary variable, considering generalized TC seizures and seizures with secondary generalization as a group, and the other non-convulsive seizures as reference group.

Patients presenting convulsive seizures and patients with more than 5 seizures/month were the most drug-resistant, probably because they were also the most active and severe cases. Adults appeared more drug-resistant than children and this could had been related to the presence among the first group of more cases of symptomatic epilepsy, as often reported in sub-Saharan Africa [6], [39], [40].

Concerning AEs, few events were recorded in our cohort showing a progressive reduction of their appearance over time, in line with that experienced in others resource-poor countries [41], [42].

A very important issue often reported in resource-poor countries is the presence of a large proportion of PWE that soon discontinues the treatment. Das et al. [43] has coined the term “secondary treatment gap” to designate this phenomenon highlighting that the main reason of AEDs discontinuation could be found in some socio-economic factors, mainly identified with the inability to afford the treatment and with the lack of information about the consequences of medication noncompliance. In the first year of treatment we pointed out a rate of withdrawals of 26.0%, considering all people lost to follow-up. This definition does not exactly correspond to an effective treatment discontinuation. Our cohort, in fact, was mainly constituted by male workers traveling to different places for seasonal field works and, therefore, it could be possible that some withdrawals have continued elsewhere the treatment. Nevertheless, in the attempt to better understand the characteristics of these patients, we tried to identify some predicting factors of being withdrawn. Being a woman was an important obstacle to continuing the treatment. As was elsewhere underlined [44], women encounter much more difficulty and social prejudices, as stigma and discrimination, that impede an independent access to health-care centers increasing treatment gap [9], [45]. Had never consulted a doctor for epilepsy and had never been treated, even discontinuously, with AEDs were two important factors associated with treatment discontinuation, probably linked to a better level of knowledge and information about epilepsy and its treatment. This highlight the importance of awareness campaign performed at the beginning of every treatment programs.

Patients with convulsive seizures and with a high number of seizures per month were, as mentioned before, the most drug-resistant and appeared also as more likely to discontinuing treatment, probably for reduced recovery expectation [3], [46]. However, this finding need to be further investigated as we also found that the risk of withdrawal seemed to be independent from epilepsy remission (p = 0.7).

Our findings showed that the treatments with PB presented a good efficacy, a good tolerability and a good compliance in this cohort of PWE treated at primary health-care centers. This shows how important was in the RARE intervention program, the inclusion of campaigns aimed at ameliorate both patients and health-care personnel knowledge on the disease and on the importance of a good compliance with long-term treatments. Nevertheless further efforts are still needed to assure a broader accessibility to treatment programs for the entire population (including women and children) and to improve the compliance and continuity with treatment, essential steps for the effective reduction of the burden of epilepsy in resource-poor countries.
